# *catena*-Poly[[[μ-aqua-penta­aqua­dizinc(II)]-μ_4_-benzene-1,2,4,5-tetra­carboxyl­ato] dihydrate]

**DOI:** 10.1107/S160053680902666X

**Published:** 2009-07-11

**Authors:** Archimede Rotondo, Giuseppe Bruno, Francesco Nicoló, Angelo Cento

**Affiliations:** a Dipartimento di Chimica Inorganica, Chimica Analitica e Chimica Fisica, Universitá di Messina, Salita Sperone, 31-98166-Messina, Italy; b ITCGC Ferraris, Reggio Calabria, Italy

## Abstract

The asymmetric unit of the title compound, {[Zn_2_(C_10_H_2_O_8_)(H_2_O)_6_]·2H_2_O}_*n*_, contains two distinct Zn atoms joined by a bridging water molecule and two bridging carboxyl­ate groups belonging to distinct halves of benzene-1,2,4,5-tetra­carboxyl­ate (tbec) tetra­anionic ligands, both lying on crystallographic inversion centres. The structure of this new isopolymorphic one-dimensional coordination polymer features asymmetric bimetallic octa­hedral knots. O—H⋯O hydrogen bonds between water molecules and carboxylate O atoms help to consolidate the crystal packing.

## Related literature

For background to 1,2,4,5,-benzene­tetra­carboxylate anions, see: Robl (1987 ▶); Wei *et al.* (1991 ▶). For their use in constructing stable metal-organic frameworks, see: Du *et al.* (2007 ▶); Rochon & Massarweh (2000 ▶); Wang *et al.* (2007 ▶); Wen *et al.* (2007 ▶); Yang *et al.* (2003 ▶). For a description of the Cambridge Structural Database, see: Allen (2002 ▶).
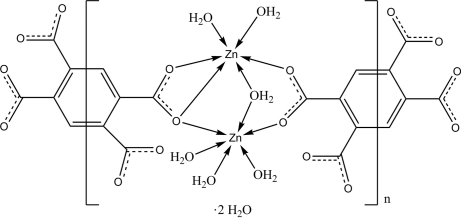


## Experimental

### 

#### Crystal data

[Zn_2_(C_10_H_2_O_8_)(H_2_O)_6_]·2H_2_O
*M* *_r_* = 525.02
Triclinic, 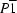

*a* = 6.8429 (1) Å
*b* = 8.0167 (1) Å
*c* = 16.6700 (2) Å
α = 101.620 (1)°
β = 92.555 (1)°
γ = 93.439 (1)°
*V* = 892.62 (2) Å^3^
*Z* = 2
Mo *K*α radiation
μ = 2.77 mm^−1^
*T* = 296 K
0.50 × 0.40 × 0.12 mm

#### Data collection

Bruker APEXII diffractometer
Absorption correction: ψ scan (North *et al.*, 1968 ▶) *T*_min_ = 0.300, *T*_max_ = 0.717
17507 measured reflections
3632 independent reflections
3506 reflections with *I* > 2σ(*I*)
*R*_int_ = 0.051

#### Refinement

*R*[*F* ^2^ > 2σ(*F* ^2^)] = 0.029
*wR*(*F* ^2^) = 0.083
*S* = 1.04
3632 reflections
279 parameters
H-atom parameters constrained
Δρ_max_ = 0.80 e Å^−3^
Δρ_min_ = −0.72 e Å^−3^

### 

Data collection: *APEX2* (Bruker, 2007 ▶); cell refinement: *SAINT* (Bruker, 2007 ▶); data reduction: *SAINT*; program(s) used to solve structure: *SHELXS97* (Sheldrick, 2008 ▶); program(s) used to refine structure: *SHELXL97* (Sheldrick, 2008 ▶); molecular graphics: *XP* (Bruker, 2007 ▶), *ORTEP-3 for Windows* (Farrugia, 1997 ▶) and *Mercury* (Macrae *et al.*, 2006 ▶); software used to prepare material for publication: WingGX (Farrugia, 1999 ▶), *PARST* (Nardelli, 1995 ▶), *enCIFer* (Allen *et al.*, 2004 ▶) and *PLATON* (Spek, 2009 ▶).

## Supplementary Material

Crystal structure: contains datablocks global, I. DOI: 10.1107/S160053680902666X/jh2083sup1.cif

Structure factors: contains datablocks I. DOI: 10.1107/S160053680902666X/jh2083Isup2.hkl

Additional supplementary materials:  crystallographic information; 3D view; checkCIF report

## Figures and Tables

**Table 1:** Selected geometric parameters (Å, °)

Zn1—O1*W*	2.0374 (15)
Zn1—O5*W*	2.0464 (16)
Zn1—O2	2.0484 (14)
Zn1—O2*W*	2.0550 (16)
Zn1—O6	2.1462 (14)
Zn1—O1*B*	2.2540 (14)
Zn2—O4*W*	1.9886 (16)
Zn2—O1	2.0065 (13)
Zn2—O3*W*	2.0525 (17)
Zn2—O5	2.0868 (13)
Zn2—O1*B*	2.1693 (14)
Zn2—O6	2.4325 (15)

**Table 2:** Hydrogen-bond geometry (Å, °)

*D*—H⋯*A*	*D*—H	H⋯*A*	*D*⋯*A*	*D*—H⋯*A*
O1*B*—H1*BA*⋯O4^i^	0.85	1.82	2.667 (2)	178
O1*B*—H1*BB*⋯O7^i^	0.85	1.79	2.638 (2)	175
O1*W*—H1*WA*⋯O8^i^	0.85	1.90	2.721 (2)	163
O1*W*—H1*WB*⋯O8^ii^	0.85	1.97	2.770 (2)	156
O2*W*—H2*WA*⋯O3^i^	0.85	1.85	2.689 (2)	171
O2*W*—H2*WB*⋯O5^iii^	0.85	1.90	2.724 (2)	163
O3*W*—H3*WB*⋯O6*W*^iv^	0.85	1.89	2.737 (2)	174
O3*W*—H3*WA*⋯O7*W*	0.85	1.98	2.829 (3)	178
O4*W*—H4*WA*⋯O3^v^	0.85	1.81	2.661 (2)	176
O4*W*—H4*WB*⋯O6*W*	0.85	1.80	2.649 (2)	175
O5*W*—H5*WA*⋯O7*W*^iii^	0.85	1.93	2.768 (2)	170
O5*W*—H5*WB*⋯O8	0.85	2.01	2.855 (2)	171
O6*W*—H6*WA*⋯O1^vi^	0.85	1.94	2.774 (2)	167
O6*W*—H6*WB*⋯O4^i^	0.85	1.91	2.687 (2)	152
O7*W*—H7*WA*⋯O3^vii^	0.85	2.15	2.995 (3)	171
O7*W*—H7*WB*⋯O7	0.85	1.89	2.721 (2)	167

Symmetry codes: (i) 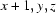; (ii) 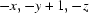; (iii) 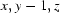; (iv) 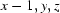; (v) 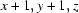; (vi) 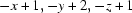; (vii) 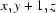.

**Fig. 1.**
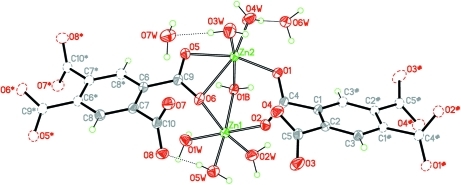
View of I. Displacement ellipsoids are drawn at the 40% probability level and dashed atoms are obtained by symmetry transformations. Symmetry codes #: -x, -y + 1, -z + 1; *: -x, -y + 2, -z.

**Fig. 2.**
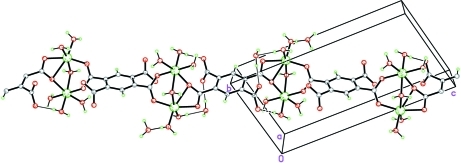
Monodimensional framework running along the long diagonal of b and c crystallographic axes. Dashed lines indicate intermolecular hydrogen bonds also reported in the tables.

## supplementary crystallographic information

### Comment

There are many crystallogaraphic studies of 1,2,4,5,-benzenetetracarboxylic ions (btec) coordinated to zinc in the Cambridge Structural Database (Allen, 2002). The previous studies (Robl, 1987; Wei *et al.*, 1991) were followed by others aimed at building stable metal-organic frameworks even exploiting hydrothermal conditions (Wen *et al.* 2007; Wang *et al.*, 2007; Rochon & Massarweh 2000; Yang *et al.* 2003; Du *et al.* 2007). The polymorph presented here is obtained from a simple water solution containing the sodium dicarboxylate, zinc nitrate and melamine. The two metal centers of (I) display a skewed octahedral geometry (Figure 1, Table 1) with water molecules supplementing the organic ligands. Beyond the mono-dimensional scaffold running along the long diagonal of the b and c crystallographic axes (Figure 2), a close hydrogen bonding network supports the crystal packing (Table 2). Similar syntheses with different amines demonstrate that the btec coordination modes and packing strongly depend on the nature of the metal and the ancillary amines used (Bruno & Rotondo, to be published)

### Experimental

A water solution of 5 ml of Zn(NO_3_)_2_ 50mM with an equimolar solution of melamine were aded to 10 ml of a 25mM disodium-dihydrogen 1,2,4,5-benzenetetracarboxylate solution. The resulting clear solution with pH= 5.15 was left covered at room temperature Colourless crystals could be separated from the solution after five days.

### Refinement

All hydrogen atoms were located in the difference map and refined in ideal positions with the 'riding and rigid model' technique. Temperature factors are always related to the parent atoms.

### Crystal data

**Table:**

[Zn_2_(C_10_H_2_O_8_)(H_2_O)_6_]·2H_2_O	*Z* = 2
*M**_r_* = 525.02	*F*(000) = 532
Triclinic, *P*1	*D*_x_ = 1.953 Mg m^−^^3^
Hall symbol: -P 1	Mo *K*α radiation, λ = 0.71073 Å
*a* = 6.8429 (1) Å	Cell parameters from 3632 reflections
*b* = 8.0167 (1) Å	θ = 4.1–26.4°
*c* = 16.6700 (2) Å	µ = 2.77 mm^−^^1^
α = 101.620 (1)°	*T* = 296 K
β = 92.555 (1)°	Laminar, colourless
γ = 93.439 (1)°	0.5 × 0.4 × 0.12 mm
*V* = 892.62 (2) Å^3^	

### Data collection

**Table:**

Bruker APEXII diffractometer	3506 reflections with *I* > 2σ(*I*)
graphite	*R*_int_ = 0.051
ω scans	θ_max_ = 26.4°, θ_min_ = 4.1°
Absorption correction: ψ scan (North *et al.*, 1968)	*h* = −8→8
*T*_min_ = 0.3, *T*_max_ = 0.717	*k* = −10→10
17507 measured reflections	*l* = −20→20
3632 independent reflections	

### Refinement

**Table:**

Refinement on *F*^2^	Primary atom site location: structure-invariant direct methods
Least-squares matrix: full	Secondary atom site location: difference Fourier map
*R*[*F*^2^ > 2σ(*F*^2^)] = 0.029	Hydrogen site location: inferred from neighbouring sites
*wR*(*F*^2^) = 0.083	H-atom parameters constrained
*S* = 1.04	*w* = 1/[σ^2^(*F*_o_^2^) + (0.0561*P*)^2^ + 0.4217*P*] where *P* = (*F*_o_^2^ + 2*F*_c_^2^)/3
3632 reflections	(Δ/σ)_max_ = 0.001
279 parameters	Δρ_max_ = 0.80 e Å^−^^3^
0 restraints	Δρ_min_ = −0.72 e Å^−^^3^

### Special details

**Table:**

Geometry. All e.s.d.'s (except the e.s.d. in the dihedral angle between two l.s. planes) are estimated using the full covariance matrix. The cell e.s.d.'s are taken into account individually in the estimation of e.s.d.'s in distances, angles and torsion angles; correlations between e.s.d.'s in cell parameters are only used when they are defined by crystal symmetry. An approximate (isotropic) treatment of cell e.s.d.'s is used for estimating e.s.d.'s involving l.s. planes.
Refinement. Refinement of *F*^2^ against ALL reflections. The weighted *R*-factor *wR* and goodness of fit *S* are based on *F*^2^, conventional *R*-factors *R* are based on *F*, with *F* set to zero for negative *F*^2^. The threshold expression of *F*^2^ > σ(*F*^2^) is used only for calculating *R*-factors(gt) *etc*. and is not relevant to the choice of reflections for refinement. *R*-factors based on *F*^2^ are statistically about twice as large as those based on *F*, and *R*- factors based on ALL data will be even larger.

### Fractional atomic coordinates and isotropic or equivalent isotropic displacement parameters (Å^2^)

**Table:**

	*x*	*y*	*z*	*U*_iso_*/*U*_eq_	
Zn1	0.19779 (3)	0.58919 (3)	0.188584 (13)	0.02614 (10)	
Zn2	0.26320 (3)	0.96925 (3)	0.309777 (13)	0.02578 (9)	
C1	0.0467 (3)	0.5880 (2)	0.43868 (11)	0.0224 (3)	
C2	−0.1459 (3)	0.5219 (2)	0.44205 (11)	0.0221 (3)	
C3	−0.1898 (3)	0.4336 (2)	0.50353 (11)	0.0236 (3)	
H3	−0.3173	0.3884	0.5059	0.028*	
C4	0.1070 (3)	0.6707 (2)	0.36925 (11)	0.0235 (3)	
C5	−0.3060 (3)	0.5493 (2)	0.38211 (11)	0.0250 (4)	
O1	0.1889 (2)	0.82096 (17)	0.38882 (8)	0.0292 (3)	
O2	0.0801 (2)	0.58438 (19)	0.29902 (8)	0.0333 (3)	
O3	−0.4229 (3)	0.4248 (2)	0.35058 (12)	0.0491 (5)	
O4	−0.3135 (2)	0.69436 (19)	0.36674 (10)	0.0365 (4)	
C6	0.0639 (3)	0.9763 (2)	0.07689 (11)	0.0238 (3)	
C7	−0.1212 (3)	0.9066 (2)	0.04319 (11)	0.0240 (4)	
C8	−0.1827 (3)	0.9309 (2)	−0.03354 (12)	0.0260 (4)	
H9	−0.3053	0.8844	−0.0565	0.031*	
C9	0.1354 (3)	0.9520 (2)	0.15918 (11)	0.0244 (4)	
C10	−0.2624 (3)	0.8139 (2)	0.08949 (12)	0.0249 (4)	
O5	0.2597 (2)	1.06218 (18)	0.20148 (8)	0.0293 (3)	
O6	0.0691 (2)	0.82610 (18)	0.18669 (9)	0.0292 (3)	
O7	−0.3423 (2)	0.90212 (19)	0.14747 (9)	0.0330 (3)	
O8	−0.2988 (2)	0.65586 (18)	0.06365 (9)	0.0325 (3)	
O1B	0.4460 (2)	0.77301 (17)	0.25175 (8)	0.0258 (3)	
H1BA	0.5201	0.7459	0.2887	0.039*	
H1BB	0.519	0.8101	0.2183	0.039*	
O1W	0.3101 (2)	0.5950 (2)	0.07794 (9)	0.0350 (3)	
H1WA	0.4347	0.6028	0.0811	0.052*	
H1WB	0.2724	0.5135	0.0382	0.052*	
O2W	0.3691 (3)	0.39693 (19)	0.20722 (11)	0.0391 (4)	
H2WA	0.429	0.4152	0.2543	0.055 (9)*	
H2WB	0.3207	0.2942	0.1968	0.062 (10)*	
O3W	0.0456 (3)	1.1285 (2)	0.34625 (11)	0.0429 (4)	
H3WB	−0.0281	1.0929	0.3799	0.064*	
H3WA	−0.0278	1.1538	0.3086	0.064*	
O4W	0.4683 (3)	1.1212 (2)	0.38193 (13)	0.0457 (4)	
H4WA	0.4971	1.2194	0.3721	0.069*	
H4WB	0.5726	1.0857	0.4002	0.069*	
O5W	−0.0407 (2)	0.4428 (2)	0.12882 (10)	0.0361 (3)	
H5WA	−0.1	0.3807	0.1571	0.054*	
H5WB	−0.1224	0.5086	0.1144	0.054*	
O6W	0.7863 (3)	1.0157 (2)	0.44733 (9)	0.0365 (3)	
H6WA	0.793	1.0501	0.4991	0.055*	
H6WB	0.7683	0.9075	0.4373	0.055*	
O7W	−0.1929 (3)	1.2210 (2)	0.22149 (12)	0.0436 (4)	
H7WA	−0.268	1.2784	0.254	0.065*	
H7WB	−0.2568	1.1285	0.1981	0.065*	

### Atomic displacement parameters (Å^2^)

**Table:**

	*U*^11^	*U*^22^	*U*^33^	*U*^12^	*U*^13^	*U*^23^
Zn1	0.03275 (15)	0.02292 (14)	0.02313 (14)	−0.00024 (10)	0.00215 (10)	0.00610 (9)
Zn2	0.03256 (15)	0.02316 (14)	0.02257 (14)	−0.00172 (10)	0.00053 (10)	0.00829 (9)
C1	0.0280 (9)	0.0191 (8)	0.0207 (8)	−0.0007 (7)	0.0026 (7)	0.0060 (6)
C2	0.0258 (8)	0.0203 (8)	0.0199 (8)	0.0002 (6)	−0.0015 (6)	0.0044 (6)
C3	0.0240 (8)	0.0239 (8)	0.0232 (8)	−0.0020 (7)	0.0009 (7)	0.0068 (7)
C4	0.0243 (8)	0.0237 (8)	0.0243 (9)	0.0005 (7)	−0.0001 (7)	0.0096 (7)
C5	0.0268 (9)	0.0267 (9)	0.0225 (8)	0.0007 (7)	−0.0013 (7)	0.0084 (7)
O1	0.0401 (8)	0.0230 (6)	0.0250 (6)	−0.0055 (5)	0.0033 (6)	0.0081 (5)
O2	0.0456 (8)	0.0312 (7)	0.0224 (7)	−0.0100 (6)	0.0034 (6)	0.0070 (5)
O3	0.0503 (10)	0.0357 (8)	0.0617 (11)	−0.0184 (7)	−0.0307 (9)	0.0244 (8)
O4	0.0439 (9)	0.0244 (7)	0.0411 (8)	0.0001 (6)	−0.0145 (7)	0.0105 (6)
C6	0.0278 (9)	0.0233 (8)	0.0211 (8)	0.0005 (7)	0.0009 (7)	0.0068 (6)
C7	0.0263 (9)	0.0222 (8)	0.0246 (9)	−0.0003 (7)	0.0036 (7)	0.0070 (7)
C8	0.0257 (9)	0.0272 (9)	0.0251 (9)	−0.0033 (7)	0.0004 (7)	0.0072 (7)
C9	0.0262 (9)	0.0251 (8)	0.0242 (9)	0.0049 (7)	0.0035 (7)	0.0086 (7)
C10	0.0256 (9)	0.0275 (9)	0.0230 (9)	−0.0017 (7)	0.0004 (7)	0.0096 (7)
O5	0.0349 (7)	0.0284 (7)	0.0246 (6)	−0.0025 (6)	−0.0049 (6)	0.0083 (5)
O6	0.0315 (7)	0.0286 (7)	0.0313 (7)	0.0028 (5)	0.0038 (6)	0.0149 (6)
O7	0.0371 (8)	0.0303 (7)	0.0322 (7)	−0.0014 (6)	0.0125 (6)	0.0069 (6)
O8	0.0361 (8)	0.0274 (7)	0.0326 (7)	−0.0079 (6)	0.0045 (6)	0.0052 (6)
O1B	0.0254 (6)	0.0278 (7)	0.0264 (7)	0.0008 (5)	0.0028 (5)	0.0106 (5)
O1W	0.0358 (8)	0.0386 (8)	0.0274 (7)	−0.0063 (6)	0.0053 (6)	0.0013 (6)
O2W	0.0450 (9)	0.0261 (7)	0.0447 (9)	0.0010 (6)	−0.0083 (7)	0.0071 (6)
O3W	0.0494 (10)	0.0450 (9)	0.0390 (9)	0.0158 (8)	0.0111 (7)	0.0144 (7)
O4W	0.0499 (10)	0.0279 (8)	0.0581 (11)	−0.0095 (7)	−0.0232 (8)	0.0149 (7)
O5W	0.0387 (8)	0.0319 (8)	0.0370 (8)	−0.0044 (6)	−0.0034 (7)	0.0091 (6)
O6W	0.0466 (9)	0.0305 (7)	0.0312 (7)	0.0025 (7)	−0.0010 (7)	0.0045 (6)
O7W	0.0473 (10)	0.0316 (8)	0.0506 (10)	−0.0004 (7)	0.0074 (8)	0.0055 (7)

### Geometric parameters (Å, °)

**Table:**

Zn1—O1W	2.0374 (15)	C6—C9	1.489 (2)
Zn1—O5W	2.0464 (16)	C7—C8	1.383 (3)
Zn1—O2	2.0484 (14)	C7—C10	1.514 (2)
Zn1—O2W	2.0550 (16)	C8—C6^ii^	1.390 (3)
Zn1—O6	2.1462 (14)	C8—H9	0.93
Zn1—O1B	2.2540 (14)	C9—O6	1.259 (2)
Zn2—O4W	1.9886 (16)	C9—O5	1.268 (2)
Zn2—O1	2.0065 (13)	C10—O7	1.245 (3)
Zn2—O3W	2.0525 (17)	C10—O8	1.259 (2)
Zn2—O5	2.0868 (13)	O1B—H1BA	0.85
Zn2—O1B	2.1693 (14)	O1B—H1BB	0.8499
Zn2—O6	2.4325 (15)	O1W—H1WA	0.85
Zn2—C9	2.5955 (19)	O1W—H1WB	0.8499
C1—C3^i^	1.386 (3)	O2W—H2WA	0.85
C1—C2	1.398 (3)	O2W—H2WB	0.8499
C1—C4	1.506 (2)	O3W—H3WB	0.85
C2—C3	1.391 (3)	O3W—H3WA	0.8499
C2—C5	1.506 (2)	O4W—H4WA	0.85
C3—C1^i^	1.386 (3)	O4W—H4WB	0.8499
C3—H3	0.93	O5W—H5WA	0.85
C4—O2	1.234 (2)	O5W—H5WB	0.8499
C4—O1	1.271 (2)	O6W—H6WA	0.85
C5—O4	1.243 (2)	O6W—H6WB	0.8499
C5—O3	1.252 (2)	O7W—H7WA	0.85
C6—C8^ii^	1.390 (3)	O7W—H7WB	0.8499
C6—C7	1.399 (3)		
			
O1W—Zn1—O5W	89.16 (6)	O4—C5—C2	117.88 (17)
O1W—Zn1—O2	178.96 (7)	O3—C5—C2	118.09 (17)
O5W—Zn1—O2	90.13 (6)	C4—O1—Zn2	125.40 (12)
O1W—Zn1—O2W	92.28 (7)	C4—O2—Zn1	135.19 (13)
O5W—Zn1—O2W	98.80 (6)	C8^ii^—C6—C7	120.02 (17)
O2—Zn1—O2W	88.58 (7)	C8^ii^—C6—C9	119.49 (17)
O1W—Zn1—O6	89.74 (6)	C7—C6—C9	120.50 (16)
O5W—Zn1—O6	93.96 (6)	C8—C7—C6	119.02 (17)
O2—Zn1—O6	89.55 (6)	C8—C7—C10	118.37 (17)
O2W—Zn1—O6	167.10 (6)	C6—C7—C10	122.52 (17)
O1W—Zn1—O1B	90.00 (5)	C7—C8—C6^ii^	120.96 (17)
O5W—Zn1—O1B	174.33 (6)	C7—C8—H9	119.5
O2—Zn1—O1B	90.63 (5)	C6^ii^—C8—H9	119.5
O2W—Zn1—O1B	86.83 (6)	O6—C9—O5	120.91 (17)
O6—Zn1—O1B	80.43 (5)	O6—C9—C6	120.18 (17)
O4W—Zn2—O1	97.62 (7)	O5—C9—C6	118.89 (16)
O4W—Zn2—O3W	93.08 (8)	O6—C9—Zn2	68.41 (10)
O1—Zn2—O3W	91.65 (7)	O5—C9—Zn2	52.68 (9)
O4W—Zn2—O5	103.69 (7)	C6—C9—Zn2	169.69 (13)
O1—Zn2—O5	158.69 (6)	O7—C10—O8	124.97 (18)
O3W—Zn2—O5	87.18 (6)	O7—C10—C7	117.16 (17)
O4W—Zn2—O1B	98.94 (7)	O8—C10—C7	117.73 (17)
O1—Zn2—O1B	88.79 (6)	C9—O5—Zn2	98.43 (11)
O3W—Zn2—O1B	167.80 (7)	C9—O6—Zn1	128.94 (12)
O5—Zn2—O1B	87.99 (5)	C9—O6—Zn2	82.82 (11)
O4W—Zn2—O6	160.35 (7)	Zn1—O6—Zn2	91.75 (5)
O1—Zn2—O6	101.22 (5)	Zn2—O1B—Zn1	96.19 (5)
O3W—Zn2—O6	91.92 (6)	Zn2—O1B—H1BA	108.5
O5—Zn2—O6	57.60 (5)	Zn1—O1B—H1BA	120
O1B—Zn2—O6	76.05 (5)	Zn2—O1B—H1BB	110.8
O4W—Zn2—C9	132.49 (7)	Zn1—O1B—H1BB	112.9
O1—Zn2—C9	129.83 (6)	H1BA—O1B—H1BB	107.7
O3W—Zn2—C9	88.13 (6)	Zn1—O1W—H1WA	112
O5—Zn2—C9	28.89 (6)	Zn1—O1W—H1WB	117.1
O1B—Zn2—C9	82.25 (5)	H1WA—O1W—H1WB	107.7
O6—Zn2—C9	28.77 (5)	Zn1—O2W—H2WA	114.1
C3^i^—C1—C2	119.98 (17)	Zn1—O2W—H2WB	119.7
C3^i^—C1—C4	118.49 (16)	H2WA—O2W—H2WB	107.7
C2—C1—C4	121.35 (17)	Zn2—O3W—H3WB	113
C3—C2—C1	118.96 (17)	Zn2—O3W—H3WA	116.9
C3—C2—C5	119.88 (17)	H3WB—O3W—H3WA	107.7
C1—C2—C5	121.13 (16)	Zn2—O4W—H4WA	118.5
C1^i^—C3—C2	121.06 (17)	Zn2—O4W—H4WB	123.4
C1^i^—C3—H3	119.5	H4WA—O4W—H4WB	107.7
C2—C3—H3	119.5	Zn1—O5W—H5WA	114.7
O2—C4—O1	125.95 (17)	Zn1—O5W—H5WB	108.5
O2—C4—C1	117.27 (16)	H5WA—O5W—H5WB	107.7
O1—C4—C1	116.71 (16)	H6WA—O6W—H6WB	107.7
O4—C5—O3	124.03 (18)	H7WA—O7W—H7WB	107.7
			
C3^i^—C1—C2—C3	−0.5 (3)	O3W—Zn2—C9—C6	50.3 (7)
C4—C1—C2—C3	174.41 (16)	O5—Zn2—C9—C6	−37.3 (7)
C3^i^—C1—C2—C5	177.60 (17)	O1B—Zn2—C9—C6	−137.2 (7)
C4—C1—C2—C5	−7.5 (3)	O6—Zn2—C9—C6	147.7 (8)
C1—C2—C3—C1^i^	0.5 (3)	C8—C7—C10—O7	−104.6 (2)
C5—C2—C3—C1^i^	−177.61 (17)	C6—C7—C10—O7	71.8 (2)
C3^i^—C1—C4—O2	117.8 (2)	C8—C7—C10—O8	71.4 (2)
C2—C1—C4—O2	−57.2 (3)	C6—C7—C10—O8	−112.2 (2)
C3^i^—C1—C4—O1	−59.3 (2)	O6—C9—O5—Zn2	−5.4 (2)
C2—C1—C4—O1	125.70 (19)	C6—C9—O5—Zn2	172.89 (14)
C3—C2—C5—O4	134.3 (2)	O4W—Zn2—O5—C9	176.27 (12)
C1—C2—C5—O4	−43.8 (3)	O1—Zn2—O5—C9	−3.9 (2)
C3—C2—C5—O3	−46.0 (3)	O3W—Zn2—O5—C9	−91.24 (13)
C1—C2—C5—O3	135.9 (2)	O1B—Zn2—O5—C9	77.56 (12)
O2—C4—O1—Zn2	8.8 (3)	O6—Zn2—O5—C9	2.83 (10)
C1—C4—O1—Zn2	−174.40 (12)	O5—C9—O6—Zn1	−81.8 (2)
O4W—Zn2—O1—C4	−157.51 (16)	C6—C9—O6—Zn1	99.93 (19)
O3W—Zn2—O1—C4	109.16 (16)	Zn2—C9—O6—Zn1	−86.43 (13)
O5—Zn2—O1—C4	22.7 (3)	O5—C9—O6—Zn2	4.61 (17)
O1B—Zn2—O1—C4	−58.65 (16)	C6—C9—O6—Zn2	−173.65 (16)
O6—Zn2—O1—C4	16.87 (16)	O1W—Zn1—O6—C9	−35.77 (16)
C9—Zn2—O1—C4	20.22 (19)	O5W—Zn1—O6—C9	−124.92 (16)
O1—C4—O2—Zn1	19.5 (3)	O2—Zn1—O6—C9	144.99 (16)
C1—C4—O2—Zn1	−157.33 (15)	O2W—Zn1—O6—C9	63.3 (3)
O5W—Zn1—O2—C4	−161.3 (2)	O1B—Zn1—O6—C9	54.27 (16)
O2W—Zn1—O2—C4	99.9 (2)	O1W—Zn1—O6—Zn2	−117.95 (5)
O6—Zn1—O2—C4	−67.4 (2)	O5W—Zn1—O6—Zn2	152.91 (6)
O1B—Zn1—O2—C4	13.1 (2)	O2—Zn1—O6—Zn2	62.81 (5)
C8^ii^—C6—C7—C8	0.4 (3)	O2W—Zn1—O6—Zn2	−18.8 (3)
C9—C6—C7—C8	−179.71 (17)	O1B—Zn1—O6—Zn2	−27.91 (4)
C8^ii^—C6—C7—C10	−176.03 (18)	O4W—Zn2—O6—C9	−22.1 (2)
C9—C6—C7—C10	3.9 (3)	O1—Zn2—O6—C9	174.66 (11)
C6—C7—C8—C6^ii^	−0.4 (3)	O3W—Zn2—O6—C9	82.59 (11)
C10—C7—C8—C6^ii^	176.18 (18)	O5—Zn2—O6—C9	−2.84 (10)
C8^ii^—C6—C9—O6	−155.20 (18)	O1B—Zn2—O6—C9	−99.44 (11)
C7—C6—C9—O6	24.9 (3)	O4W—Zn2—O6—Zn1	106.93 (19)
C8^ii^—C6—C9—O5	26.5 (3)	O1—Zn2—O6—Zn1	−56.30 (6)
C7—C6—C9—O5	−153.42 (18)	O3W—Zn2—O6—Zn1	−148.36 (6)
C8^ii^—C6—C9—Zn2	59.9 (8)	O5—Zn2—O6—Zn1	126.20 (7)
C7—C6—C9—Zn2	−120.0 (7)	O1B—Zn2—O6—Zn1	29.61 (5)
O4W—Zn2—C9—O6	170.11 (11)	C9—Zn2—O6—Zn1	129.05 (12)
O1—Zn2—C9—O6	−6.83 (14)	O4W—Zn2—O1B—Zn1	171.17 (6)
O3W—Zn2—C9—O6	−97.42 (11)	O1—Zn2—O1B—Zn1	73.64 (6)
O5—Zn2—C9—O6	175.03 (18)	O3W—Zn2—O1B—Zn1	−18.6 (3)
O1B—Zn2—C9—O6	75.05 (11)	O5—Zn2—O1B—Zn1	−85.29 (5)
O4W—Zn2—C9—O5	−4.91 (16)	O6—Zn2—O1B—Zn1	−28.23 (4)
O1—Zn2—C9—O5	178.14 (11)	C9—Zn2—O1B—Zn1	−56.86 (5)
O3W—Zn2—C9—O5	87.55 (13)	O1W—Zn1—O1B—Zn2	121.58 (6)
O1B—Zn2—C9—O5	−99.97 (12)	O2—Zn1—O1B—Zn2	−57.59 (6)
O6—Zn2—C9—O5	−175.03 (18)	O2W—Zn1—O1B—Zn2	−146.14 (7)
O4W—Zn2—C9—C6	−42.2 (8)	O6—Zn1—O1B—Zn2	31.84 (5)
O1—Zn2—C9—C6	140.9 (7)		

Symmetry codes: (i) −*x*, −*y*+1, −*z*+1; (ii) −*x*, −*y*+2, −*z*.

### Hydrogen-bond geometry (Å, °)

**Table:**

*D*—H···*A*	*D*—H	H···*A*	*D*···*A*	*D*—H···*A*
O1B—H1BA···O4^iii^	0.85	1.82	2.667 (2)	178
O1B—H1BB···O7^iii^	0.85	1.79	2.638 (2)	175
O1W—H1WA···O8^iii^	0.85	1.90	2.721 (2)	163
O1W—H1WB···O8^iv^	0.85	1.97	2.770 (2)	156
O2W—H2WA···O3^iii^	0.85	1.85	2.689 (2)	171
O2W—H2WB···O5^v^	0.85	1.90	2.724 (2)	163
O3W—H3WB···O6W^vi^	0.85	1.89	2.737 (2)	174
O3W—H3WA···O7W	0.85	1.98	2.829 (3)	178
O4W—H4WA···O3^vii^	0.85	1.81	2.661 (2)	176
O4W—H4WB···O6W	0.85	1.80	2.649 (2)	175
O5W—H5WA···O7W^v^	0.85	1.93	2.768 (2)	170
O5W—H5WB···O8	0.85	2.01	2.855 (2)	171
O6W—H6WA···O1^viii^	0.85	1.94	2.774 (2)	167
O6W—H6WB···O4^iii^	0.85	1.91	2.687 (2)	152
O7W—H7WA···O3^ix^	0.85	2.15	2.995 (3)	171
O7W—H7WB···O7	0.85	1.89	2.721 (2)	167

Symmetry codes: (iii) *x*+1, *y*, *z*; (iv) −*x*, −*y*+1, −*z*; (v) *x*, *y*−1, *z*; (vi) *x*−1, *y*, *z*; (vii) *x*+1, *y*+1, *z*; (viii) −*x*+1, −*y*+2, −*z*+1; (ix) *x*, *y*+1, *z*.
